# Photothermal raster image correlation spectroscopy of gold nanoparticles in solution and on live cells

**DOI:** 10.1098/rsos.140454

**Published:** 2015-06-17

**Authors:** D. J. Nieves, Y. Li, D. G. Fernig, R. Lévy

**Affiliations:** 1 Department of Biochemistry, Institute of Integrative Biology, University of Liverpool, Biosciences Building, Crown Street, Liverpool L69 7ZB, UK; 2 EMBL Australia Node in Single Molecule Science, Lowy Cancer Research Centre, University of New South Wales, Sydney, NSW 2052, Australia

**Keywords:** fluctuation spectroscopy, diffusion, PHI, RICS, gold nanoparticles, FGF2

## Abstract

Raster image correlation spectroscopy (RICS) measures the diffusion of fluorescently labelled molecules from stacks of confocal microscopy images by analysing correlations within the image. RICS enables the observation of a greater and, thus, more representative area of a biological system as compared to other single molecule approaches. Photothermal microscopy of gold nanoparticles allows long-term imaging of the same labelled molecules without photobleaching. Here, we implement RICS analysis on a photothermal microscope. The imaging of single gold nanoparticles at pixel dwell times short enough for RICS (60 μs) with a piezo-driven photothermal heterodyne microscope is demonstrated (photothermal raster image correlation spectroscopy, PhRICS). As a proof of principle, PhRICS is used to measure the diffusion coefficient of gold nanoparticles in glycerol : water solutions. The diffusion coefficients of the nanoparticles measured by PhRICS are consistent with their size, determined by transmission electron microscopy. PhRICS was then used to probe the diffusion speed of gold nanoparticle-labelled fibroblast growth factor 2 (FGF2) bound to heparan sulfate in the pericellular matrix of live fibroblast cells. The data are consistent with previous single nanoparticle tracking studies of the diffusion of FGF2 on these cells. Importantly, the data reveal faster FGF2 movement, previously inaccessible by photothermal tracking, and suggest that inhomogeneity in the distribution of bound FGF2 is dynamic.

## 1. Introduction

The direct observation of individual molecules by optical microscopy [1] in living cells [2–6] allows access to movement, fluctuations, colocalization and conformational changes at the molecular level, all of which are critical features of molecular function. A variety of optical microscopy techniques have been developed to probe this molecular heterogeneity, providing insights into biological processes, from the compartmentalization of the plasma membrane [7] to dynamics and stoichiometry of epidermal growth factor receptor complex formation [8]. Of particular biological relevance, and studied for many years (see [9] for an early example), the diffusion of biomolecules has been investigated via positional tracking or analyses of temporal and spatial correlations.

Single molecule tracking (SMT) employs fluorescent labels such as fluorescent dyes or fusion proteins, whereas single particle tracking (SPT) uses nanoparticles [10–15]. The observation of fluorescent labels, e.g. proteins or dyes, is limited by photobleaching [16], while longer sequences can be acquired by SPT, as detection is based on scattering or absorption, e.g. gold nanoparticles [10,11], which are completely stable over time. The data are in the form of a ‘track’ of the observed molecule, which contains information on a broad range of diffusion modes/coefficients. Analysis of the tracks has provided novel insights into the heterogeneity of the molecules' environment and interactions [17–19]. One example is the organization of the cell membrane gained by SPT of transmembrane receptors labelled with gold nanoparticles [9,10], e.g. the compartmentalization of the cell membrane, as a mechanism to modulate membrane movement and function [9,10]. Both SMT and SPT, to effectively probe the biological system, require a large number of tracks to provide statistical power to the measurements [17,18,20]. Often implicit in these studies is an assumption of representation, i.e. although the tracks only cover a fraction of a cell, they are representative of the entire biological system. To improve coverage over larger areas on single cells within a single experiment, Giannone *et al.* [20] introduced universal point accumulation imaging in the nanoscale topography (uPAINT), which uses constant imaging during cell labelling to generate a super-resolution image based on short (before photobleaching) single molecule tracks.

Correlation spectroscopy includes single point detection, i.e. fluorescence correlation spectroscopy (FCS) [21–24] and raster imaging correlation spectroscopy [25]. Similar to SMT and SPT, FCS has limitations in terms of coverage of the biological system [22,26]. In particular, slow molecules are lost either due to photobleaching within the detection volume or because of their low probability to diffuse through this volume within the duration of the experiment. The consequence is a biased representation of the biological system, with an over representation of fast-moving molecules [27]. Image correlation spectroscopy (ICS) has the advantage of providing a better spatial coverage and is appropriate for observing clustering of slowly diffusing components, for example, molecules embedded in or bound to the membrane (diffusion coefficient, *D*, approx. 0.1 μm^2^ s^−1^) [28]. ICS can, therefore, provide a diffusion map, but it is limited to slowly diffusing events on the timescale of milliseconds to seconds (image frame rate). To bridge the time resolution gap between single point FCS and ICS, Digman *et al.* [25] introduced an extension to these techniques known as fluorescent raster image correlation spectroscopy (RICS). RICS can be applied to most existing fluorescent laser scanning microscopes, as it exploits the intrinsic time structure of raster scan images. The analysis provides temporal information in the range of: microseconds (a pixel or many pixels), milliseconds (a scan line or many scan lines) and seconds (an image or multiple images) [25]. This, coupled with the spatial information within the image enables ‘fast’ (microsecond) and ‘slow’ (second) dynamics of biomolecules to be probed over a relatively large detection area. RICS has already proved a useful tool for investigating the diffusion and concentration of fluorophores in solution and fluorescently labelled proteins and molecules in cells [25,29–31]. In its initial application, RICS was used to measure enhanced green fluorescent protein (EGFP) in solution as a proof of principle, and then applied to probe a fluorescent protein-tagged adhesion protein, paxillin, in Chinese hamster ovary cells. The probing of multiple areas of the cell revealed that where focal adhesions were present the diffusion coefficient of paxillin was much lower than when observed in the cytoplasm away from the adhesions [25]. The approach has since been applied and extended for a variety of measurements such as the measurement of non-isotropic movement of fluorescently labelled ATP in cardiomyocytes [31] and the observation of bovine serum albumin aggregation in denaturing conditions [32].

Photothermal heterodyne imaging (PHI) provides highly sensitive detection of gold nanoparticles [18,33–36]. PHI relies on the lock-in detection of scattering around an absorbing nanoparticle, owing to the heat released from its surface under excitation at its plasmon resonance [37,38]. PHI modalities encompass both single nanoparticle tracking [34] and correlation spectroscopy [39–42]. Photothermal absorption correlation spectroscopy (PhACS) exploits the photothermal signal to allow the observation of gold nanoparticles, and the molecules they label in solution [39–41], and can observe a wide range of diffusion speeds. However, the data acquired by PhACS, like FCS, lacks spatial and temporal information about the distribution of the probe, owing to the small sampling volume (approx. femtolitre) [39,40]. Here, we extend the RICS approach to PHI (photothermal raster image correlation spectroscopy, PhRICS) to bridge between photothermal tracking and PhACS measurements by simultaneously observing the spatial distribution of nanoparticles and extracting diffusion dynamics at a wide range of timescales. We present, as a proof of principle, measurements made on gold nanoparticles in solution. The method is then applied to probe the diffusion of a gold nanoparticle-labelled protein, fibroblast growth factor 2 (FGF2), in the pericellular matrix of live fibroblast cells.

## 2. Material and methods

### 2.1 Preparation of single 8.8 nm gold nanoparticle samples using poly-l-lysine

A rectangular coverslip (22×44 mm, Leica Surgipath, Leica Microsystems, Milton Keynes, UK) was incubated with poly-l-lysine solution (MW 70 000–15 0000 Da; 0.01% (v/v), Sigma Aldrich, Gillingham, UK) for 40 min to adhere. The coverslip was washed three times with Milli-Q ultrapure water. A 1–2 pM solution of 8.8 nm gold nanoparticles (nominally 10 nm, BBI Solutions Ltd., Cardiff, UK) was then added to the coverslip and left for another 40 min. The coverslip was washed again three times with Milli-Q water and mounted in 80% glycerol (glycerol: Fisher Scientific, Leicester, UK). A second coverslip was added on top with a Parafilm ‘M’ spacer, and the chamber sealed by melting the parafilm with a soldering iron.

### 2.2 Preparation of 8.8 nm gold nanoparticle samples in glycerol : water

Nanoparticles (as above) were diluted in the appropriate glycerol : water mixture (indicated in text). The solution was introduced by capillarity into a homemade fluidic channel formed with Parafilm ‘M’ between two coverslips.

### 2.3 Synthesis of 8.8 nm CVVVT-ol : HS-C_11_-EG_4_-OH (70 : 30) capped nanoparticles

Nanoparticles (as above) were capped with a mixture of peptidol (CVVVT-ol) and alkanethiol ethyleneglycol (HS-C_11_-EG_4_-OH) in a 70 : 30 proportion as described previously by Duchesne *et al*. [43]. Briefly, 8.8 nm gold nanoparticles were mixed with the 2 mM CVVVT-ol : HS-C_11_-EG_4_-OH (70 : 30) ligand mix in a ratio of nine parts gold nanoparticles to one part ligand mix. One-tenth volume of phosphate-buffered saline (10× PBS: 1.4 M NaCl, 27 mM KCl, 81 mM Na_2_PO_4_, 12 mM KH_2_PO_4_, pH 7.4) supplemented with Tween-20 0.1% (v/v) was added as buffer after the addition of ligands. This mixture was left on a wheel with mixing overnight. Excess ligands were removed by G-25 Sephadex size-exclusion chromatography with 1× PBS supplemented with 0.005% (v/v) Tween-20 as the mobile phase.

### 2.4 Nanoparticle functionalization with FGF proteins (1 : 1)

The synthesis and subsequent conjugation of FGF2 protein to maleimide nanoparticles was previously described by Nieves *et al.* [44]. Briefly, maleimide functionalized gold nanoparticles were incubated with a 35 times molar excess of recombinant FGF2 (produced according to Ke *et al*. [45]) for 3 h at room temperature. The conjugation of FGF2 to the maleimide functionalized gold nanoparticle proceeds via thiol-Michael addition with the thiol side chain of the exposed cysteine (C-95) at the surface of the FGF2 (FGF2-NP). The mixture was then centrifuged for 80 min at 13 000 *g* and 4°C. The supernatant containing the excess FGF2 was removed and the particles were resuspended in 200 μl of 1× PBS Tween-20 0.005% (v/v). The centrifugation was performed four times in order to remove all excess FGF2, and the pellet was finally resuspended in 1× PBS Tween-20 0.005% (v/v).

### 2.5 Cell culture

Rat mammary (Rama 27) fibroblast cells were cultured on plastic in Dulbecco's modified Eagle's medium (DMEM, Life Technologies, Paisley, UK) supplemented with 10% (v/v) fetal calf serum, 50 ng ml^−1^ insulin, 50 ng ml^−1^ hydrocortisone and 0.05% (v/v) sodium bicarbonate (all from Life Technologies) at 37°C in 10% (v/v) CO_2_ [46]. When required for photothermal imaging, cells were seeded onto a rectangular coverslip (22×44 mm, Leica Surgipath) and left overnight. The cells were then incubated for 2 h in step-down medium (DMEM with 250 μg ml^−1^ bovine serum albumin (BSA, Sigma Aldrich, cat no. 9048-46-8) and used for live cell experiments.

### 2.6 Preparation of live cell FGF-NP experiments

Live Rama 27 cells seeded onto coverslips in step-down medium were washed three times with PBS. They were then incubated with 600 pM FGF2-NP for 1 h, after which the cells were washed again three times with PBS to remove unbound FGF2-NP. The cells were mounted in Krebs Ringer buffer (pH 7.4; 10 mM HEPES, 140 mM NaCl, 5 mM KCl, 2 mM CaCl_2_, 2 mM MgCl_2_, 11 mM glucose), with a second coverslip on top for photothermal imaging and heat-sealed using Parafilm ‘M’ spacer.

### 2.7 PHI set-up

**Figure 1.**
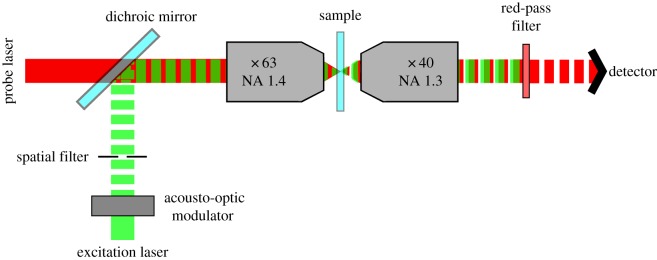
Photothermal microscope set-up used for photothermal imaging and PhRICS.

All images were acquired using a homebuilt photothermal confocal microscope (a schematic is presented in figure 1). The excitation laser (523 nm; frequency-doubled ND : YAG, Ventus Laser Quantum, Germany) was modulated at a frequency of 459.5 kHz using an acousto-optical modulator (Isomet Corporation, UK). This excitation beam was ‘cleaned’ using a spatial filter. This was done to remove ellipticity generated by the modulation and generates a Gaussian beam profile. Excitation laser power for all imaging was 2 mW. The excitation beam was overlaid with a non-resonant probe laser (633 nm, 10 mW; JDS Uniphase Corporation) via a cold mirror (ThorLabs). The superimposed beams were focused onto the sample via an oil immersion objective (Zeiss Plan-Apochromat 63×, numerical aperture (NA) 1.4). The sample was placed on a piezo scanning stage (MCL502385, MadCity Labs, Madison, WI, USA), which allows movement of the sample in three dimensions (*x*, *y* and *z*) over the fixed laser spot. Scanning by a piezoelectric stage driver (MCL NanoDrive 85, USA) under the control of the Nanonis RC4 module and Nanonis program (Specs-Zurich, Zurich, Switzerland) was used to move the sample over the fixed laser spot. The transmitted and forward scattered light was collected by a second oil objective (Zeiss NEOFLUAR 40×, NA 1.3) and passed through a red-pass filter (ThorLabs) to block the excitation laser. The red component was focused upon one photodiode of the balanced photoreceiver (Model 2107 10 MHz adjustable photoreceiver, New Focus, USA). A lock-in amplifier (DSP 7260, Signal Recovery, Oak Ridge, TN, USA) was used to identify the scattered component of the probe beam that corresponds to the modulation frequency or ‘beat-note’ (i.e. 459.5 kHz). A Nanonis SC4 Acquisition Module (Specs-Zurich) was used for signal acquisition. The signal was averaged (pixel dwell time indicated in text) and a greyscale pixel value was generated. The values along a scan path, i.e. photothermal signal intensity at each position, were then converted into a photothermal image. The images were saved in a .sxm format.

### 2.8 Acquisition parameters for PhRICS

The sample was raster scanned across the detection volume multiple times and the images saved. The images were 160×128 pixels with a pixel size of 50 nm (thus, the ROI was 8.0×6.4 μm) and a pixel dwell time of 60 μs. A rectangular image was taken to allow settling of the stage at the beginning of the scan line. In addition, the return of the stage to the next scan line after acquisition of the previous occurred at the same speed of acquisition. Thus, the time per line is double that expected for a single scan line, i.e. 19.2 ms not 9.6 ms. At least 40 images were acquired for RICS analysis. For imaging of fixed nanoparticles, the bottom objective was focused at the coverslip. For imaging of diffusion in solution, it was focused 8–10 μm into solution, whereas for live cell experiments it was focused 1 μm above the coverslip.

### 2.8.1 Lateral dimension of the detection volume (1/*e*^2^ radius)

Images of 8.8 nm gold nanoparticles immobilized on poly-l-lysine were taken using the PhRICS imaging parameters. The images were converted from .sxm to .txt files using Gwyddion (http://gwyddion.net/) [47]. The images were then processed using ImageJ (http://imagej.nih.gov/ij/) to give the final 128×128 pixel PhRICS image and saved as a 16-bit tiff image. Line profiles of the peaks were fitted with a Gaussian curve using Origin 8.6. From the fit, the full width at half maximum (FWHM) was derived and the 1/*e*^2^ radius calculated using the following equation;

$$2w=\frac{\sqrt{2}\mathrm{FWHM}}{\sqrt{\ln2}},$$

where *w* is the full width of the volume at 1/*e*^2^.

### 2.9 Analysis of PhRICS data

Images were first converted from .sxm to .txt files using Gwyddion. The images were then were cropped, i.e. 32 pixels removed at the beginning of each scan line, using ImageJ to give the final 128×128 pixel PhRICS image and saved as a 16-bit tiff image sequence. All the images that underpin the results presented here are available through Figshare (see Data accessibility). The sequence was then loaded into the RICS module of the SimFCS software [48]. Firstly, a moving average of the image sequence (a window of 10 images) was taken. The moving average was subtracted from the image sequence to remove any immobile features from the images, i.e. signal that persists at the same position throughout the image sequence (described by Digman *et al.* [25]), and the spatial autocorrelation function applied. The surface of the resulting autocorrelation image was then fitted using the SimFCS program [48] with the following fixed parameters: time per line (19.2 ms), 1/*e*^2^ radius of the detection volume (0.240 μm) and pixel size (50 nm). The fit resulted in extraction of the diffusion coefficient for mobile features in the image sequence.

## 3. Results and discussion

### 3.1 Imaging parameters for PhRICS

**Figure 2.**
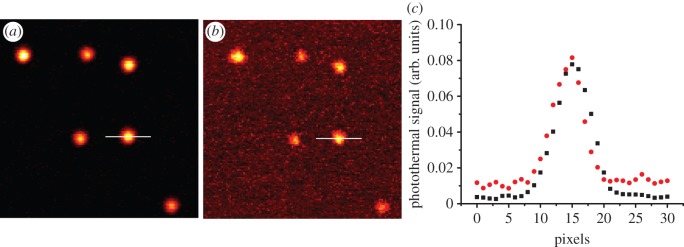
PHI and detection of single 8.8 nm gold nanoparticles. (*a*) Image acquired with 1 ms pixel dwell time. (*b*) Image acquired with 60 μs pixel dwell time. (c) Comparison of the photothermal signal profiles of the same gold nanoparticle in (*a*) (squares) and (*b*) (circles). Both images are 6.4×6.4 μm with a pixel size of 50 nm.

Typically, PHI of gold nanoparticles uses pixel dwell times of the order of 1–10 ms [18,49–51]. Correlation spectroscopy both of fluorescent and non-fluorescent objects [25,39–41] indicate that shorter dwell times, of the order of microseconds, are required. Thus, we first evaluated whether such pixel dwell times would still provide sufficient signal-to-noise ratio (SNR). Encouragingly, PHI imaging with a pixel dwell time of 80 μs has been achieved recently using a galvanometric laser scanning system [52]. In most PHI systems, including our own, a piezoelectric stage is used for scanning [38,50,53]. Thus, the sample is moved over a fixed laser spot, as opposed to the galvanometric method that moves the laser spot over the sample. While piezo-scanning simplifies laser alignment, speed is limited by stage stability and response time. Photothermal images of single 8.8 nm gold nanoparticles immobilized on poly-l-lysine acquired using pixel dwell times of 1 ms and 60 μs were acquired (figure 2*a*,*b*). Single 8.8 nm gold nanoparticles were detected at both 1 ms and 60 μs dwell times (figure 2*a*,*b*). However, for 60 μs pixel dwell times the imaging area had to be extended in the scan direction (to 8.0 μm and cropped to show the same area, as described §2.8) to allow for settling of the stage after every scan line. Comparison of the photothermal signal profile of the same nanoparticle (figure 2*c*) showed that the peak pixel value is very similar, but the SNR goes from over 200 to approximately 10 when the dwell time is reduced from 1 ms to 60 μs (SNR was calculated by dividing the average photothermal signal of the peak by the standard deviation of the background noise). This SNR is high enough to be used for single nanoparticle tracking [34], thus, the acquisition of rapid raster scan images with a piezo-stage PHI microscope for RICS analysis is possible.

RICS fitting of spatial correlations requires knowledge of the 1/*e*^2^ radius of the detection volume. Therefore, images of single gold nanoparticles were acquired with the same parameters, as in figure 2*b*, and the FWHM of the curve derived by Gaussian fitting. The 1/*e*^2^ was then calculated from the FWHM and was found to be 240±24 nm (*n*=23).

### 3.2 Determination of the diffusion coefficient of gold nanoparticles in solution by PhRICS

**Figure 3.**
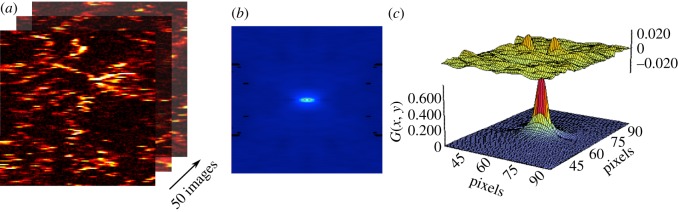
Workflow for PhRICS experiment. (*a*) Consecutive rapid raster scan images are acquired of the sample. (*b*) The spatial correlation for each image in the stack is then calculated using SimFCS software and then the average of all the correlations taken. (*c*) The surface plot of the spatial correlation is then fitted with the model for the diffusion of molecules during a raster scan.

**Figure 4.**
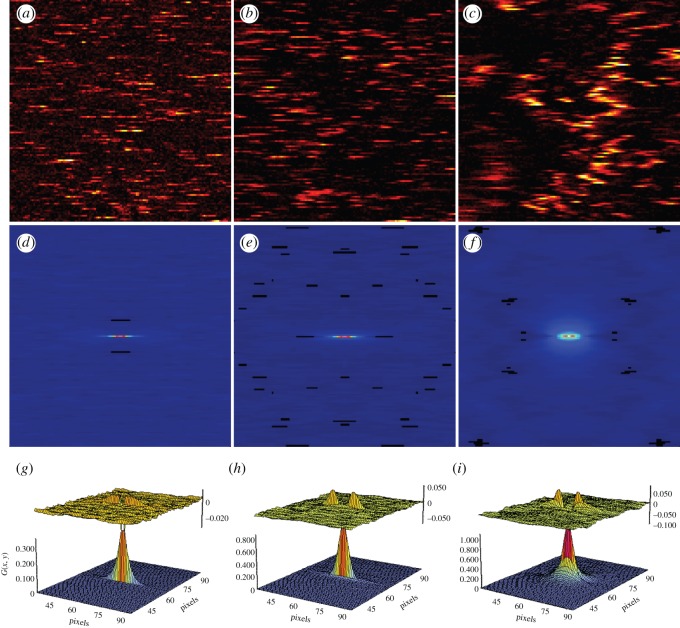
PhRICS of 8.8 nm gold nanoparticles in different glycerol : water mixtures. Nanoparticles in 20% (v/v) glycerol (*a*,*d*,*g*), 50% glycerol (*b*,*e*,*h*) and 80% glycerol (*c*,*f*,*i*) were imaged by PhRICS (*a*–*c*) and the corresponding average spatial correlation for nanoparticles was calculated (*d*–*f*), as well as fitting result for each spatial correlation (bottom surface; *g*–*i*). The differences between the fitting model and data for *g*–*i* are also plotted (top surface).

The rationale for the RICS methodology has been extensively discussed [25,29,54], and the ability to extract information on diffusion dynamics from raster scan images has demonstrated its power for probing the movement of biomolecules in live cells [29,30]. Initially, the technique was verified by measurements performed upon samples of molecules/objects of known diffusion coefficients as a validation of the method. For example, RICS was able to effectively measure the diffusion speed and concentration of fluorescein and monomeric EGFP in solution, which compared favourably with FCS measurements of the same sample [29,55]. To determine whether accurate diffusion coefficients of nanoparticles can be extracted by PhRICS, 8.8 nm gold nanoparticles were suspended in glycerol : water (v/v) of different viscosities (20%, 50% and 80%, all v/v). Similar conditions have been used previously to establish autocorrelation spectroscopy of gold nanoparticles [39,41]. The experimental workflow for PhRICS is presented in figure 3. Exemplar images and the resulting spatial correlation and fitting are shown in figure 4. The diffusion of nanoparticles through the detection volume during the raster scan results in a characteristic ‘streaking’ pattern, which has previously been observed for mobile nanoparticle-labelled molecules [18,34]. As glycerol concentration (and, therefore, viscosity) was increased (figure 4*a*–*c*), the number and length of streaks observed per line decreased, and the persistence of gold nanoparticles from one scan line to the next became more common, which is consistent with a reduction in diffusion speed. The correlation properties described qualitatively above are reflected in the spatial correlations for each glycerol concentration (figure 4*d*–*f*), which decrease in length along the *x*-direction and begin to become broader in the *y*-direction. PhRICS measurements were performed on three different samples for each glycerol concentration and the extracted diffusion coefficients from the fitting (figure 4*g*–*i*) were averaged (table 1; all reduced *χ*^2^-values for the fits were below 0.00008). The diameter distribution of the 8.8 nm gold nanoparticles was determined by transmission electron microscopy (TEM) to be 8.8±1.1 nm (*n*=2604). Thus, the expected *D* for these nanoparticles in different glycerol concentrations can be calculated using the Stokes–Einstein equation. The expected *D* range calculated in table 1 corresponded to plus and minus 1 s.d. Potentially, photothermal heating of nanoparticles can lead to an effect known as ‘hot Brownian motion’, whereby the increase in surface temperature can cause an increase in diffusion speed [41]. This effect was reported previously for nanoparticles of diameters down to 40 nm. Owing to the difference in the thermal conductivity, and thus the potential nanoparticle surface temperature rise, we have calculated the temperature profiles generated for 8.8 nm gold nanoparticles in this set-up (electronic supplementary material, figure S1). The calculated surface temperature increases for 20%, 50% and 80% glycerol (all v/v) are 19.9, 25.1 and 32.2 K, respectively. The measured *D* values by PhRICS for each glycerol concentration were in good agreement with the range expected, and it appears that the diffusion of 8.8 nm gold nanoparticles is not thermally enhanced at this range of surface temperatures. The corresponding hydrodynamic diameter of the nanoparticles observed for 20%, 50% and 80% glycerol are 9.5±1.0 nm, 9.6±0.9 nm and 9.2±0.7 nm. The slight increase in diameter as compared from TEM value might result from the observation of clusters of few nanoparticles during the PhRICS acquisition. Owing to the intensity of these clusters being higher than the monodisperse population, it is possible that they can contribute to the final average correlation function, as has been reported before for ICS measurements [56].

**Table 1.** The measured diffusion coefficient and hydrodynamic diameter by PhRICS of 8.8±1.1 nm gold nanoparticles in different % glycerol : water mixtures.

glycerol % (v/v)	expected *D* (μm^2^ s^−1^)	measured *D* (μm^2^ s^−1^)
20	29.7–38.2	32.7±4.0
50	9.2–11.8	9.5±0.9
80	1.0–1.3	1.1±0.1

### 3.3 Probing the diffusion of gold nanoparticle-labelled FGF2 proteins on live fibroblast cells

**Figure 5.**
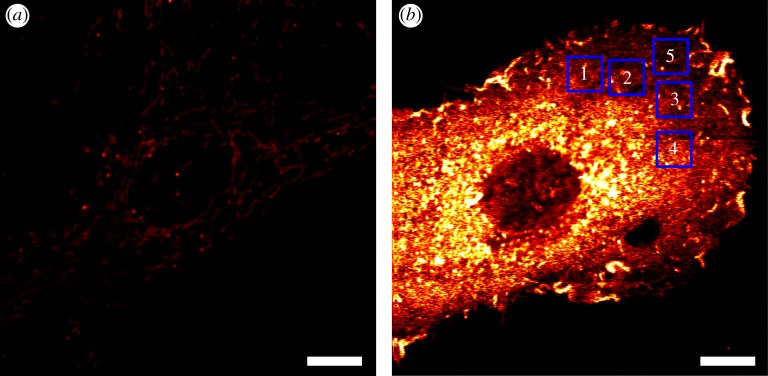
Labelling of live Rama 27 with 600 pM FGF2-NP. (*a*) Photothermal image of Rama 27 cell incubated with 600 pM nanoparticles bearing no FGF2. (*b*) Photothermal image of Rama 27 incubated with 600 pM FGF2-NP. Blue boxes indicate 6.4×6.4 μm areas that were probed by PhRICS. Scale bar, 10 μm.

**Figure 6.**
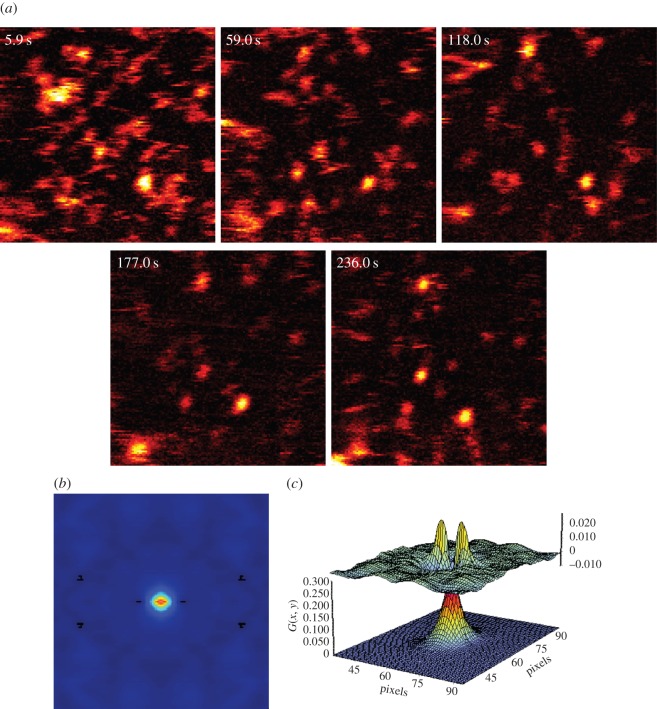
PhRICS images and analysis from box 5 (figure 5*b*). (*a*) PhRICS images from different time points throughout the acquisition. (*b*) Average spatial correlation of PhRICS stack for box 5. (*c*) Result of fitting for the spatial correlation in (*b*) (bottom surface), with the differences of the data from the model (top surface).

As an exemplar application of PhRICS, we measured the diffusion of FGF2 protein in the extracellular matrix of live rat mammary fibroblast cells (Rama 27). This system has been analysed previously by photothermal tracking [18], and FGF2 was observed to have a heterogeneous diffusion behaviour, which was attributed to the heterogeneous distribution of its binding sites on the glycosaminoglycan heparan sulfate in the pericellular matrix. Recombinant FGF2 protein was labelled with gold nanoparticles (FGF2-NP), at a stoichiometry of one FGF2 protein to one nanoparticle, as described previously [44]. When Rama 27 cells are incubated with control nanoparticles bearing no FGF2 (described in §2.3), i.e. nanoparticles that do not possess the reactive group required for FGF2 conjugation, no photothermal signal from the nanoparticles is seen (figure 5*a*). The only weak signal is that from the mitochondria present within the cell, which are detected without the need for labelling in PHI [18,57]. However, when incubated with 600 pM FGF2-NP, a very strong signal is detected (figure 5*b*). This signal is localized to the pericellular matrix of the Rama 27 cells, as FGF2 protein binds to heparan sulfate [18]. Stacks of PhRICS images were acquired in different areas of the cell membrane, away from the perinuclear region where mitochondrial signal is often high (boxes 1–5 in figure 5*b*). For all the areas observed in figure 5*b*, diffusion of FGF2-NPs was observed and the deduced diffusion coefficient for each box is presented in table 2. Here, it can be seen that there is indeed variability in the measured diffusion coefficient depending on the area being probed (table 2; reduced *χ*^2^-values for the fits range from 0.000009 to 0.000021). The diffusion coefficients range from 0.03 to 0.28 μm^2^ s^−1^. The PhRICS data and analysis from box 5 are presented in figure 6, as an example. Firstly, FGF2-NPs movement during image acquisition is indicated by the streaking pattern observed (figure 6*a*), which is consistent with previous studies [18]. There are also FGF2-NPs that do not move within a single image, as indicated by a circular intensity profile. These would not contribute to the RICS analysis, as they are treated as an ‘immobile’ feature and removed by the moving average that is subtracted from all the images in the stacks before applying the spatial correlation. These features are seen in all the areas observed in figure 5, with distribution being variable in those areas. The spatial correlation of the image stack acquired from this area (figure 6*b*) was notably broadened in the *y*-direction, as opposed to the other spatial correlations shown within this paper (figure 4*d*,*e* and *i*). This is consistent with increased correlation from one scan line to another, which could be a reflection of the confined movement observed previously of FGF2 in the pericellular matrix [18]. Fitting of the spatial correlation from box 5 yielded a diffusion coefficient of 0.16 μm^2^ s^−1^ (figure 6*c* and table 2). A comparison of the values from table 2 with those measured previously by photothermal tracking [18] demonstrates added insights provided by PhRICS into molecular movements. As noted previously, photothermal tracking has a time resolution of the order of milliseconds and cannot access very fast movement [25]. Indeed, in the data presented in the electronic supplementary material, table S2 from Duchesne *et al*. [18], only approximately 4% of the mobile fraction of FGF2 has a diffusion coefficient comparable to the values measured here by PhRICS (boxes 1, 2, 4 and 5, table 2). This suggests that the number of ‘fast’ FGF2-NPs is considerably greater than previously measured by photothermal tracking [18].

**Table 2.** The measured diffusion coefficient by PhRICS of 600 pM FGF2-NP on live Rama 27 cells from boxes 1–5 (figure 5*b*).

box from figure 5*b*	measured *D* (μm^2^ s^−1^)
1	0.14
2	0.28
3	0.03
4	0.20
5	0.16

## 4. Conclusion

The development of a new photothermal imaging technique for probing the diffusion dynamics of gold nanoparticles, PhRICS, is described. Imaging of single gold nanoparticles at short pixel dwell times (60 μs) with an SNR of 10 is achieved with a piezo-scanning PHI microscope. The ability to take images at this speed enables RICS analysis. Imaging of nanoparticles of a known size in mediums of varying viscosity confirms that quantitative measurements of diffusion coefficients across a wide dynamic range can be obtained. The method was then applied in the setting of a live cell, whereby the diffusion of gold nanoparticle-labelled FGF2 protein was explored. PhRICS was able to effectively extract diffusion measurements of FGF2 on live cells, showing that the fraction of mobile FGF2 in the pericellular matrix of fibroblasts is likely to be greater than previously determined. This has important repercussions for our understanding of cell communication and demonstrates the potential for the investigation of the movement and diffusion of proteins labelled by gold nanoparticles in live cells by PhRICS.

## Supplementary Material

A single figure (Fig. S1) with a calculation of the surface temperatures of 8.8 nm gold nanoparticles in different water:glycerol solutions under constant photothermal excitation.

## Data accessibility

All the datasets supporting this article are available at Figshare using the following doi addresses.

Figure 2:

http://dx.doi.org/10.6084/m9.figshare.1217644

Figure 4:

http://dx.doi.org/10.6084/m9.figshare.1217608

http://dx.doi.org/10.6084/m9.figshare.1217606

http://dx.doi.org/10.6084/m9.figshare.1217520

http://dx.doi.org/10.6084/m9.figshare.1217518

http://dx.doi.org/10.6084/m9.figshare.1217517

http://dx.doi.org/10.6084/m9.figshare.1217519

http://dx.doi.org/10.6084/m9.figshare.1217516

http://dx.doi.org/10.6084/m9.figshare.1217515

http://dx.doi.org/10.6084/m9.figshare.1217511

Figures 5 and 6:

http://dx.doi.org/10.6084/m9.figshare.1237096

http://dx.doi.org/10.6084/m9.figshare.1217436

http://dx.doi.org/10.6084/m9.figshare.1217631

http://dx.doi.org/10.6084/m9.figshare.1217630

http://dx.doi.org/10.6084/m9.figshare.1217629

http://dx.doi.org/10.6084/m9.figshare.1217628
