# Supplementary figures and images for: Photothermal raster image correlation spectroscopy of gold nanoparticles in solution and on live cells
Source: R Soc Open Sci. 2015 Jun 17;2(6):140454. doi: 10.1098/rsos.140454 (PMC4632534; doi:10.1098/rsos.140454)

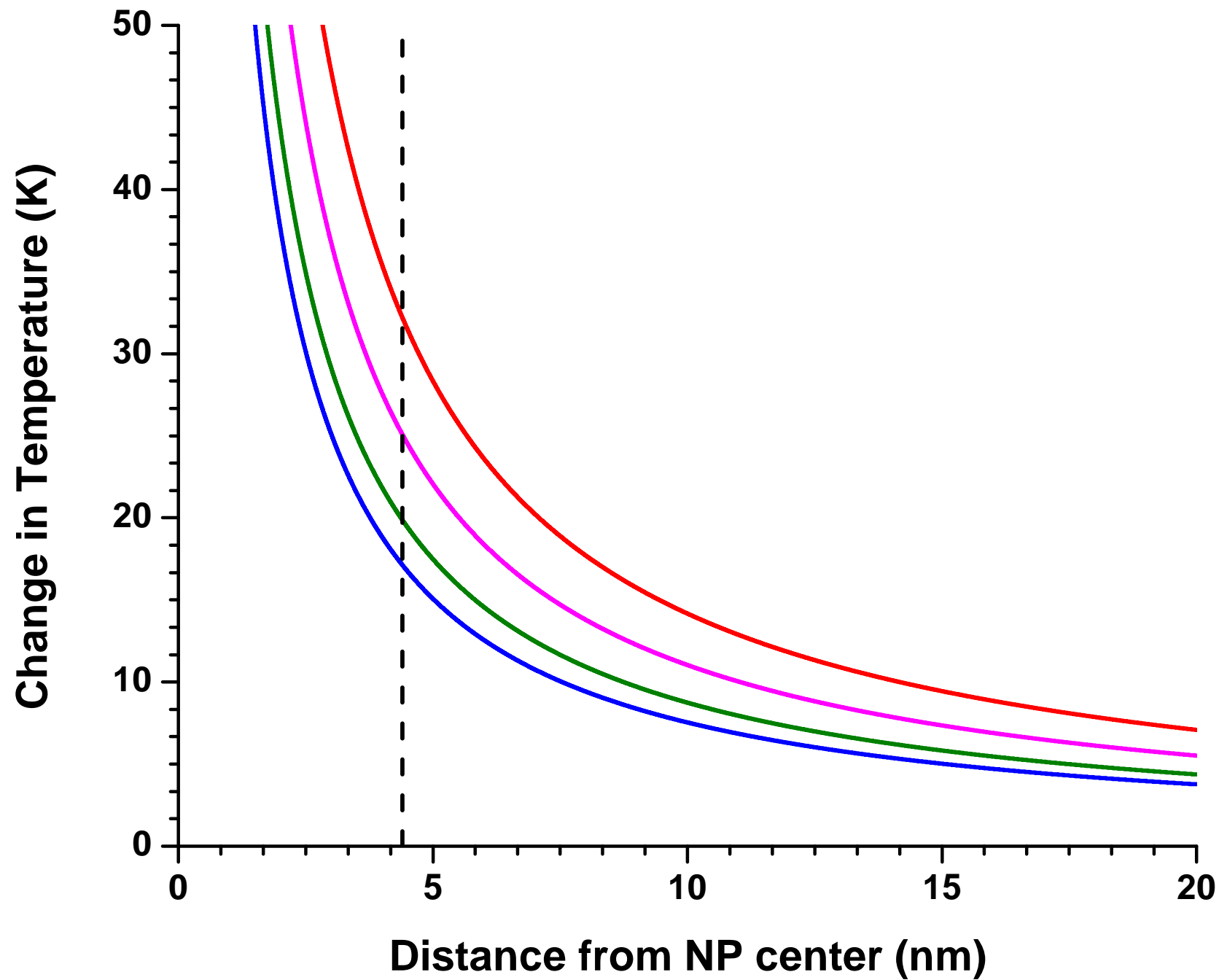

Supplement: A single figure (Fig. S1) with a calculation of the surface temperatures of 8.8 nm gold nanoparticles in different water:glycerol solutions under constant photothermal excitation. [file rsos140454supp1.pdf]
